# Language models in digital psychiatry: challenges with simplification of healthcare materials

**DOI:** 10.1038/s44277-025-00029-w

**Published:** 2025-05-22

**Authors:** Ankit Aich, Tingting Liu, Salvatore Giorgi, Kelsey Isman, Ruhshana Bobojonova, Lyle Ungar, Brenda Curtis

**Affiliations:** 1 National Institute on Drug Abuse, Baltimore, 21224 MD USA; 2 University of Pennsylvania, Philadelphia, 10587 PA USA

**Keywords:** Patient education, Health occupations

## Abstract

Linguistic hurdles in healthcare, such as complex language, significantly affect patient outcomes, including satisfaction with interaction, comprehension of healthcare materials, and engagement with the healthcare system. Reducing these hurdles has been a focus in healthcare delivery, as they significantly hinder patient engagement and adherence to treatments. The growing use of large language models (LLMs) in healthcare opens the possibility to reduce these linguistic hurdles. This study evaluates the ability of five prominent LLMs—GPT-3.5, GPT-4, GPT-4o, LLaMA-3, and Mistral—to simplify healthcare information to the standard recommended by the American Journal of Medicine. Our results indicate that while LLMs can approximate targeted reading levels, their outputs are inconsistent, with significant variability in reading level and deviation from the topic, making them unsuitable for deployment in healthcare settings.

## Lay Summary

In this work we aim to see if public-facing healthcare materials can be simplified using Large Language Models (LLMs). Currently, the American Journal of Medicine recommends that healthcare materials be provided to people at a reading level of 6. In this work we take five state of the art LLMs viz. GPT-3.5, GPT-4, GPT-4o, LLaMA-3, and Mistral-7b and experiment with prompt engineering to see if these models can simplify healthcare materials from different sources such as academic venues, CDC and WHO releases or public releases from bodies like Mayo Clinic. We find significant variability, shown through large standard-deviations in the performance of LLMs. This work paves the pathway to develop and nurture better simplification and summarization pipelines in healthcare.

## Language models in digital psychiatry: challenges with simplification of healthcare materials

Take a Deep Dive into this article

## Introduction

Linguistic hurdles, such as the complex language of healthcare materials, hinder accessibility and understanding and are well-documented challenges in health informatics and digital medicine. These hurdles have profound and far-reaching effects, influencing patient satisfaction, adherence to psychiatric treatment regimens, and overall health outcomes. Addressing these linguistic hurdles is particularly critical in psychiatry, where clear communication can foster therapeutic engagement and improve treatment adherence [1, 2]. Significant research has addressed these hurdles to enhance patient health outcomes [2].

Language-related obstacles encompass spoken and written communication, including critical elements of linguistic style such as reading level. Addressing these factors is essential for fostering effective patient engagement in healthcare settings and to expand access to digital health materials in psychiatry. In the context of psychiatry, where clear and empathetic communication is vital for adherence to treatment regimens and therapeutic engagement, simplified language can significantly enhance patient outcomes. Because of this, medical organizations have recommended health materials and patient interactions be provided with less linguistic complexity for better understanding and overall improvement in patient health outcomes. The American Journal of Medicine recommends health materials be provided at a reading level of sixth grade for best outcomes in patient health [3]. Research shows that readers overwhelmingly prefer more straightforward writing, which helps them process more information and enhances understanding [4–6]. Patients also report better care when there is a stylistic match in how information is communicated [7].

In general, simple reading material facilitates language learning, aids individuals with cognitive impairments such as aphasia or dyslexia, and enhances patient understanding in multiple settings, leading to improved adherence to treatments across multiple medical domains, from infectious diseases and immunology [8] to general medicine [9, 10] to psychiatry [11], substance use [12]. It has been shown that patient-clinician communication needs improvement through interventions that address patient needs for communication style [13]. Additionally, simplicity of language has been shown to improve health outcomes by making content more relatable and engaging for people from different backgrounds [14]. Implementing simplified and understandable healthcare material promotes communication in multiple areas and better comprehension of the materials [15], fostering greater engagement and adherence to psychiatric care. Incorporating simplification into materials has also positively impacted fields beyond healthcare, from sociology to economics [16, 17].

Large language models (LLMs) are increasingly being used across the spectrum of mental health care [18, 19], ranging from Bipolar Disorder [20, 21], Schizophrenia [21, 22], depression [23], suicidal ideation [24], stress [25], and mixed mental health tasks [26]. However, their potential to improve patient engagement and adherence to treatments remains limited by their current inability to consistently simplify complex medical information into accessible formats. In the current landscape of LLM applications in digital health, where they interact with a patient, they have been largely confined to psychiatric and mental health related settings. As such in this section, we focus on the various ways in which LLMs have been used in psychiatric care. They are also used 1 to talk to humans and generate human-like texts [27], but challenges in readability and language alignment hinder their effectiveness in fostering clear communication and engagement in general-purpose digital healthcare settings.

Despite the increasing use of artificial intelligence (AI), generative AI, and natural language processing (NLP) in healthcare, there is a notable lack of effort aimed at creating content that effectively supports patient health outcomes. In this paper, we show how public-facing, informational healthcare materials are far more complicated than the recommended level and that simplification is a significant challenge even with the latest technology. Prior studies in text simplification using natural language processing (NLP) methods often suffer from severe limitations. For instance [28] conducted simplification experiments on a mere four texts, rendering their findings difficult to generalize. Similarly, computationally intensive approaches, such as those proposed by [29], predominantly focus on simplifying medical journals, neglecting alternative sources like online blogs which are more representative of public information consumption patterns.

As exemplified by [30], general-purpose neural text simplification efforts rely on older non-transformer models and machine translation systems, largely downplaying the advancements offered by state-of-the-art LLMs. While some research has aimed at enhancing the quality of information retention in medical simplification tools [31], these efforts rarely address the capabilities and limitations of contemporary LLMs. Other related works have explored adjacent areas such as summarizing electronic health records (EHRs) [32], facilitating doctor-patient interactions [33], identifying named entities in healthcare texts [34], or using more computationally intensive methods such as entity linking [35], but these do not directly assess public-facing medical informational materials using LLMs in the broader medical domain.

While past studies have explored text simplification this paper explores the capacity of cutting-edge large language models (LLMs) to adhere to instructions concerning control over reading levels, addressing the critical issue of style and language alignment in public-facing healthcare material. With LLMs now being ubiquitous in multiple areas, this investigation is essential. We conducted experiments with five prominent LLMs, assessing their competence in simplifying medical information from various sources, including government publications, online clinics, and academic journals. Our results indicate that while LLMs demonstrate some ability to adjust style, they exhibit significant inconsistencies in reading levels, characterized by wide standard deviations. These findings raise serious concerns about the suitability of LLM deployment in healthcare-AI contexts, including patient interactions in digital healthcare.

## Methods

In this section we describe our methods comprising of data curation, prompt selection, and finally healthcare topic simplification. Our main objective is to evaluate the effectiveness of large language models (LLMs) in simplifying complex healthcare content to the recommended reading-level of 6. Before doing this, we first need to identify an effective prompt-strategy. Specifically, we aim to identify the most effective prompting strategy for optimizing performance on the simplification task. To do this, we first conduct a prompt selection phase, described in subsection “Testing the Prompting Process”, testing different prompt formulations with GPT-4. We keep all model parameters fixed and vary only the prompt text. Once we identify the best-performing prompt, we apply it uniformly across five models, GPT-3.5, GPT-4, GPT-4o, LLaMA-3, and Mistral-7B, to carry out the simplification task. The data used for the simplification task is described in the Data subsection below.

### Data

We selected five healthcare topics of significant public health importance—Attention Deficit Hyperactivity Disorder (ADHD), Vaccinations and Immunizations, Influenza (Flu), Substance Use, and Human Immunodeficiency Virus (HIV)—drawing upon existing literature to underscore their societal relevance [36–49].

For each topic, we curated five representative documents (for example a full blog post from the CDC) from authoritative sources, including government health agencies such as the Centers for Disease Control and Prevention (CDC)^2^ and the World Health Organization (WHO)^3^, leading medical institutions like the Mayo Clinic^4^, academic journals, and high-visibility online media platforms. These serve as the foundation for subsequent text simplification tasks.

### Measuring reading level

To evaluate readability, we use the Flesch-Kincaid (FK) reading level, a widely adopted metric for assessing text complexity [5]. FK scores correspond roughly with U.S. grade levels, ranging from 1 (very easy) to 20 (very difficult). However, it is possible to have texts with an FK score of more than 20, where such texts usually contain non-English characters, such as symbols or mathematical notation, or contain extremely difficult English. Texts with scores around 6 are considered accessible for middle-school students and are widely recommended for healthcare communication [3]. We compute FK scores using the Python library *Py-Readability-Metrics*^5^. Table 1 shows the initial FK reading levels of the collected texts prior to simplification.

**Table 1:** Initial Flesch-Kincaid Reading Level of Articles for Each Topic.

Topic	Post 1	Post 2	Post 3	Post 4	Post 5
ADHD	11.4	18.9	11.9	15.5	15.8
Flu	16.5	18.3	18.3	16.9	16.4
HIV	13.0	12.1	14.1	13.1	14.2
Substance Use	18.0	27.7	15.3	15.4	16.6
Vaccine	14.3	11.3	11.2	13.9	17.3

### Testing the prompting process

Before we perform our health information simplification task, we run a smaller experiment to find the best prompting method, since LLM output is highly dependent on the input prompt (i.e., text that contains instructions on how the LLM should behave). Prompts often contain examples of the desired behavior. Such prompts are referred to as *k-shot* prompts, where *k* is the number of examples appended to the instruction. This initial experiment is done to choose the *k* which attains the best simplified output (i.e., the FK score closest to the specified reading level of 6).

Since our main objective is a simplification task, we pick five questions from grade-school reading materials^6^ have GPT-4 answer these questions to get a baseline reading level. Next, we prompt GPT-4 to answer the same questions at four grade levels (1, 3, 5, and 7). GPT-4 was chosen to test our prompting process since it was the largest commercially available model with the least variance in performance at the time of conducting these experiments (November 2024). We test three different prompting paradigms: *k* = 0 (prompting only by specifying the required FK grade level), *k* = 1 (specifying the required FK grade level with one example), and *k* = 2 (specifying the required FK grade level with two examples). We prompted GPT-4 by asking it the following questions and asking it to generate an output at the specified grade level:

- Explain parts of a plant
- What is the water cycle?
- What is pollution?
- What does the sun do?
- Explain the parts of a human body.

Table 2 shows the performance difference for GPT-4 among the three prompting method experiments. We find that prompting only by specifying the FK grade level worked better for all tasks than providing examples using one or two-shot learning. We use this best-performing prompt for our healthcare experiment.

**Table 2:** Prompt Selection Experiment.

Grade Level	*k* = 0	*k* = 1	*k* = 2
1	**2.2**	8.9	2.9
3	**3.8**	5.4	5.3
5	**6.1**	7.5	7.2
7	**7.5**	9.9	10.1

Numbers denote the best FK levels for the required grade level for GPT-4. Numbers closest to the grade level are the best.

The best performing values are shown in bold font.

### Healthcare topics simplification task

This section discusses the healthcare-topics simplification task for the LLMs. As discussed in the introduction, complex language in healthcare information disbursal remains a problem. We perform a text simplification task to mitigate this problem and understand the performance of LLMs in this context.

To perform the healthcare simplification task, we use the best performing prompt (from the previous section) and pass it as an input to the five LLMs: GPT-3.5-turbo, GPT-4, GPT-4o, LLaMA-3, Mistral-Instruct-7B. These language models were chosen for a mix of their large parameter size (Mistral-7B has 7B parameters GPT-4 has 1.8 T parameters), commercial availability (GPT-4 is closed source, whereas Llama3 is open source), and popular-usage in generative AI applications [50]. A sample input is shown below.

“You are an intelligent and adaptable writing assistant. Given the following article, simplify this to a Flesch-Kincaid grade level of 6. You must also use at least 200 words^7^ in your generated answer. **article text verbatim**”

We then measure the FK reading level of the generated outputs (described in “Methods”) using the PyPi FK Python library.

## Results

We find that prompting only by specifying the FK grade level worked better for all tasks than providing examples or using one or two-shot learning. We use the best-performing prompt for our healthcare experiment. Table 2 shows the performance difference for GPT-4 among the three prompting methods.

Table 1 shows the initial FK reading level for all 25 articles selected. As shown, the lowest reading level is close to an FK score of 11. This score is considered to be at high school reading level and is higher than what is recommended. However, most articles have a reading level of 15 + , which is much higher than recommended for healthcare information.

**Fig. 1:**
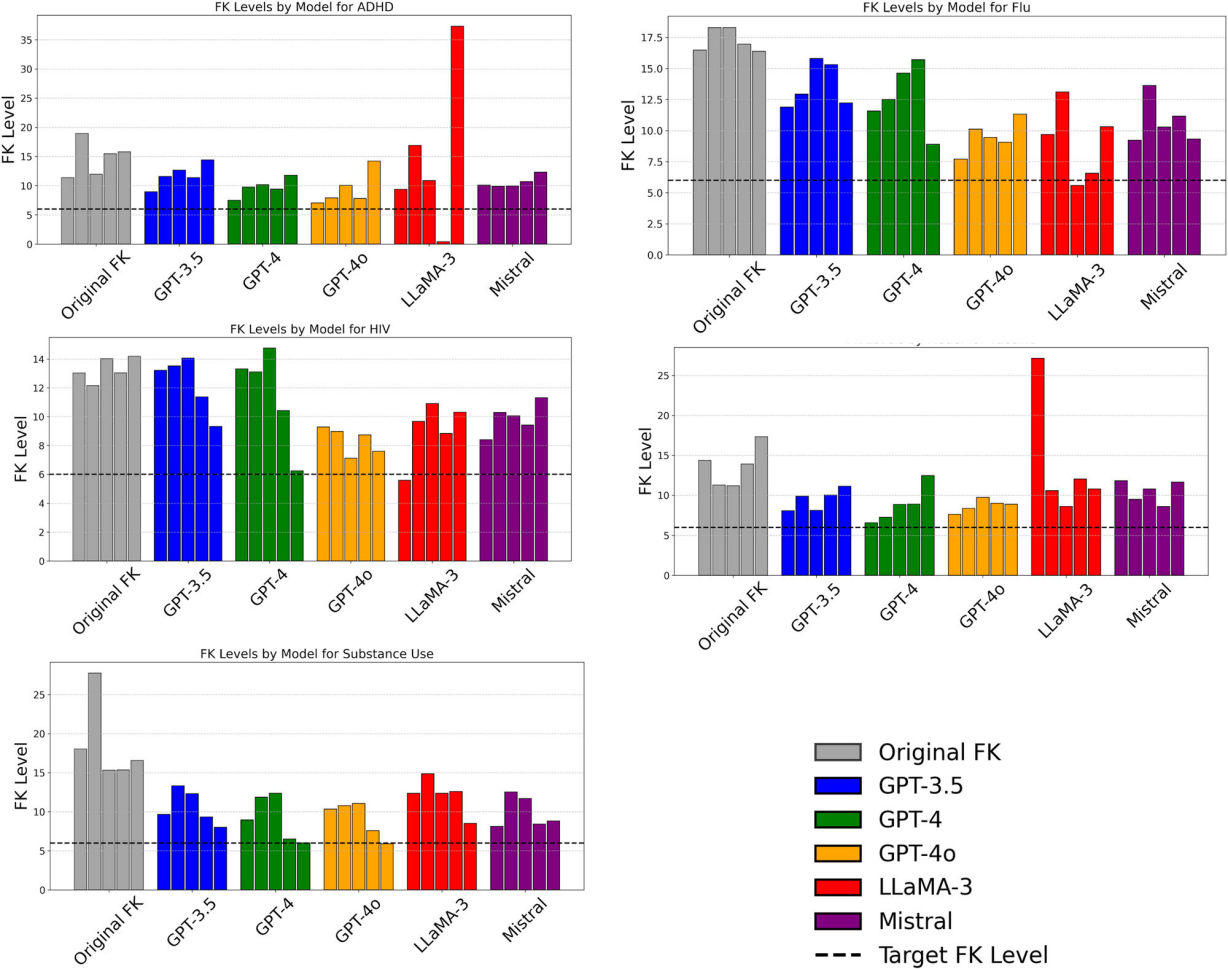
Flesch-Kincaid reading level across each topic. The dashed line indicates the ideal reading level recommended by the American Journal of Medicine [3]. The gray bars represent the reading levels of the source materials. Each bar corresponds to a specific post within the respective domain, with the bar’s position across models showing the same post’s performance across different models. For each bar cluster the bars’ position corresponds to the post number increasing left to right. Specifically, the left-most bar in a bar cluster is the FK level of post 1 and the right-most bar in a bar cluster is the FK level of post 5. Models that perform better will show reading levels closer to the dashed line than gray bars. In the above 1a - relates to vaccinations, 1b - relates to ADHD, 1c - relates to substance use, 1 d - relates to HIV, and 1e - relates to Flu.

For each of our five topics, we present results in Fig. 1 (vaccine), 2 (ADHD), 3 (substance use), 4 (HIV), 5 (flu). In each figure, the X-axis shows the models and original posts, and the Y-axis shows the FK reading levels. Each bar cluster on the X-axis comprises five bar graphs, each representing the FK level of that post. In the bar cluster, every individual bar corresponds to a post with increasing order from left to right. Specifically, the left most bar in each bar cluster is post 1, the next is post 2, and the right most bar in each bar cluster is post 5. Each graph also has the original FK level of the posts (denoted in gray), the target FK level of six (shown in a dotted line), and the outputs of five LLMs denoted by Blue for GPT-3.5, Green for GPT-4, Yellow for GPT-4o, Red for LLaMA-3, and Purple for Mistral-7B. We will discuss these graphs one by one.

As depicted in Fig. 1 (vaccines), none of the models reach the target FK reading level. While GPT-4o comes very close in two cases for posts 1 and 2, it fails to be consistent and the FK level increases for posts 3,4,5. We also see that, in general, when the baseline information has a higher initial FK level, the models struggle more. In Fig. 1 (ADHD), as shown, for the first post, all models come very close to the FK level required. In the fourth post, *LLaMA-3* reaches below the FK level in the score. However, its result is not in English (it provides a long string of text characters comprising of punctuation marks and other symbols). This artificially deflates the score but does not provide simplified information. In Fig. 1 (substance use), the models are more consistent, coming close to the required level in posts 1, 4, and 5. For HIV and flu not only are the models unable to reach the required grade level, but they also complicate the information.

We also see information in the generated text which is not related to the topic being discussed. Especially in LLaMA-3, the reading level generated is inconsistent and ranges from 0.49 to 36 when asked to generate at a sixth-grade reading level, doing worse than other models. We similarly see that the Mistral model fails to ever reach a sixth-grade level as required. This shows how some current LLMs cannot simplify or distill healthcare information at the required level of simplicity.

## Discussion

This study explores the feasibility of deploying large language models (LLMs) in digital healthcare by evaluating their ability to generate tailored text that meets specific stylistic requirements. The investigation focused on controlling output through reading level (to ensure grade-specific accessibility). Using prominent models such as GPT-3.5, GPT-4, GPT-4o, LLaMA-3, and Mistral, we examined how well these systems can adapt their language to match a required level of simplicity, denoted by FK reading level, to promote effective communication. This work provides insight into their potential role in improving healthcare communication, such as in areas of digital psychiatry, general medicine, substance use or immunology, by assessing these models’ strengths and limitations in meeting linguistic needs, which is crucial for patient engagement. We also note how bodies like the WHO and CDC do not post their findings at a required sixth-grade reading level, despite available medical recommendations and humans being able to edit text at this level. This furthers the importance of our study. Our findings highlight the opportunities and current challenges in applying LLMs to real-world, diverse healthcare settings.

Adapting the reading level of healthcare communication is essential to promote patient understanding and participation. It is widely recommended that health materials be written at a reading level of sixth to seventh grade [3] to accommodate diverse literacy needs. This study found that while some LLMs demonstrated the capability to simplify language to lower reading levels (e.g., GPT-4o achieved a minimum mean reading level of 3.2) they often struggled to achieve a low reading level over multiple iterations, as evidenced by high variability in performance (LLaMA-3 had a maximum standard deviation $\sigma$ = 27.6).

Smaller models like LLaMA-3, in particular, exhibited significant challenges, frequently generating overly complex or dissimilar language output. In general, we find that the relatively smaller LLMs, such as LLaMA-3 and Mistral, perform worse by failing to maintain consistency (defined above) and, in many cases, failing to achieve the target. The larger state-of-the-art models in the GPT family manage to stay on the topic of the question, but still fall short of achieving the required simplification target, as shown in Fig. 1 (combined graph). It is important to clarify that our observations regarding whether a model *stays on topic* were made qualitatively through manual review of the generated outputs. Specifically, we evaluated each model’s output along two dimensions: *topical relevance* and *accuracy*. We define *topical relevance* as the degree to which a response remains focused on the subject matter of the original healthcare prompt, avoiding unrelated or tangential content. *Accuracy* refers to the factual correctness and contextual appropriateness of the information provided.

While we did not quantify these patterns, the consistency of differences across multiple examples—particularly between larger and smaller models—prompted the inclusion of a qualitative assessment in the Discussion. These limitations reveal a critical gap in the ability of current large language models (LLMs) to consistently produce information in a style appropriate for healthcare, where patient comprehension directly influences treatment adherence, satisfaction, and outcomes. Addressing this challenge is essential to expanding the role of LLMs in generating effective, accessible healthcare communication. Simplifying psychiatric healthcare materials may enhance patient engagement, reduce obstacles to understanding treatment plans, and improve adherence to therapies. While prior research has explored summarizing complex texts [51–54], extracting relevant information to aid understanding [55], and supporting mental health assessments [56, 57], our findings underscore the limitations that persist in automating these simplifications reliably. This is especially relevant in mental health, infection, and immunology contexts [13], where clarity is paramount.

Adherence to digital healthcare systems is heavily influenced by the clarity and empathy of communication, especially in linguistically sensitive environments [11, 58–61]. Although LLMs show potential in simplifying healthcare content, their inconsistency in maintaining readability across outputs suggests the need for further refinements before they can reliably support patient communication. Our study highlights the difficulty of aligning automated simplifications with human-centric communication goals. Standardizing healthcare materials to simpler readability levels—such as through Flesch-Kincaid metrics—could help achieve more consistent, patient-friendly outputs. Ultimately, even though we did not directly assess adherence, improving simplification methods could foster greater comprehension and engagement, which are well-established foundations for informed decision-making and adherence to care.

This paper has several limitations. First, we conducted experiments using only five language models. Given the rapidly evolving landscape of LLMs, we focused on five widely used models that are readily accessible and possess sufficient parameter sizes to handle simplification tasks. Additionally, our healthcare simplification experiments were limited to a dataset of 25 articles. Future research could expand upon our methods by utilizing larger datasets to enhance the generalizability of the results. Future work should focus on refining LLMs by incorporating domain-specific datasets and prompts tailored for psychiatric communication, enabling more reliable and simplified patient interactions. In addition, researchers should explore integrating these models into broader digital healthcare ecosystems, such as telehealth platforms and wearable devices, to deliver more accessible and patient-centered care. Collaboration with clinical experts and patient groups will also be essential to ensure that AI tools address real-world challenges effectively and ethically. Addressing the readability challenges identified in this study can transform digital healthcare by improving patient understanding, engagement, and treatment adherence. Furthermore, developing advanced LLMs could pave the way for more all-encompassing and impactful healthcare solutions. While a huge portion of the current landscape of LLM in healthcare research has focused on psychiatry and mental health solutions, we believe stronger testing and collaboration will open them up to be used in a plethora of digital-health settings.

### Citation diversity statement

The authors have attested that they made efforts to be mindful of diversity in selecting the citations used in this article.

## Supplementary information

Excerpts for RNR

## Data Availability

Data for all articles and code can be made available by emailing the first author of the manuscript.

## Footnotes

1 https://woebothealth.com/

2 https://cdc.gov

3 https://who.int

4 https://mayoclinic.org

5 https://pypi.org/project/py-readability-metrics/

6 Sourced from K-5 Learning https://www.k5learning.com/

7 We specify 200 words because FK calculation requires at least 100 words
